# Geographical and sociodemographic disparities in fruit and vegetables consumption among adults in Burkina Faso: baseline results from the 2013 WHO STEPS survey

**DOI:** 10.1186/s12889-023-17118-0

**Published:** 2023-11-14

**Authors:** Jeoffray Diendéré, Jérôme Winbetouréfâ Somé, Jean Kaboré, Amadé Sawadogo, Estelle-Edith Dabiré, Ella Rakèta W. Compaoré, Athanase Millogo, Augustin Nawidimbasba Zeba

**Affiliations:** 1 Unit of Nutrition, Research Institute for Health Sciences (IRSS), 399, Avenue de La Liberté, 01 BP 545, Bobo-Dioulasso, Burkina Faso; 2 Ministry of Health and Public Hygiene of Burkina Faso, Ouagadougou, Burkina Faso; 3 Department of Biochemistry-Microbiology, UFR-SVT, LABIOTAN, Joseph Ki-Zerbo University, Ouagadougou, Burkina Faso; 4 Medicine Department, Sourô Sanou University Hospital, Bobo-Dioulasso, Burkina Faso

**Keywords:** Fruit and vegetables, Adequate consumption, Prevalence, Disparities, Burkina Faso

## Abstract

**Background:**

Evidence on sociodemographic determinants and spatial variations in the fruit and/or vegetable (FV) consumption was reported. This study aimed to explore geographical and sociodemographic disparities in the level of FV consumption among adults in Burkina Faso, using the national baseline data.

**Methods:**

This was a cross-sectional secondary study of primary data obtained by the 2013 (September to October) World Health Organization Stepwise Approach to Surveillance survey conducted in Burkina Faso. The participants were 4402 women and men aged 25–64 years and living in all 13 Burkinabè Regions. Descriptive and analytical analyses were performed using Student’s t test, ANOVA, the chi-square test, Fisher’s exact test and logistic regressions.

**Results:**

The prevalence of a typical daily consumption of at least three servings was 4.1% (95% CI: 3.6–4.8) for fruits and 6.6% (95% CI: 5.9–7.3) for vegetables. The national prevalence of adequate FV intake was 5.1% (95% CI: 4.4–5.8), and for two Regions (“Centre-Ouest” and “Nord”) the pooled prevalence was 22.4%, while in the other eleven Regions its was significantly lower, 2.4% (*p* = 0.0001). Using quartiles derived from the national level of consumption, each of these two Regions had a higher proportion (about 50%) of their participants in the fourth quartile (the higher level). The associated sociodemographic factors with the adequate intake were being rural residents (aOR = 1.7, *p* = 0.011) and women (aOR = 1.3; *p* = 0.03).

**Conclusion:**

Except for the Regions of “Centre-Ouest” and “Nord” of Burkina Faso, the prevalence of adequate consumption of FV was very low in its other eleven Regions. Measures to increase consumption in urban people are urgent while women should be the key actor in the family-based approaches implementation and the nutrition education promoting FV consumption.

**Supplementary Information:**

The online version contains supplementary material available at 10.1186/s12889-023-17118-0.

## Background

Non-communicable diseases (NCDs) cause a large and growing burden of morbidity and mortality in low- and middle-income countries (LMICs) [1] including Sub-Saharan Africa (SSA) countries [2]. Healthy food consumption such as adherence to a Mediterranean diet as well as the fruit and/or vegetables (FV) consumption significantly reduced overall mortality, especially related to the NCDs [3]. Systematic reviews and meta-analyses sufficiently highlighted the effectiveness of FV consumption to prevent the cardiovascular disorders [4, 5] and cancers, the most prevalent NCDs in SSA [2]. Especially, benefits of FV consumption with regard to the body fat composition, fasting insulin, blood pressure and the metabolic syndrome components were confirmed [6–8] while the low FV consumption was found among survivors from cardiovascular events in SSA [9]. The World Health Organization (WHO) recommends the implementation of a national surveillance system for the risk factors for the NCDs (stepwise approach to surveillance [*STEPS]*) including those for cardiovascular diseases and cancers [10, 11], while the US Centre for Disease Control and Prevention recommends the Behavioural Risk Factor Surveillance System [12]. The WHO STEPS surveys uses a standardized tool for data collection which includes specific section on FV consumption [10]. The survey using the STEPS method provided the first national data on FV consumption in Burkina Faso where the sampling was performed using the 13 country Regions. Sociodemographic features were relevant determinants for FV consumption [13], and respectful with the concept of the food environment, the evidence concerning spatial variations in the consumption [14, 15] is also relevant to raise, that can guide pragmatic implementation of public health interventions. Our study aimed to describe the FV consumption in a representative sample of the Burkinabè population according to socio-demographic (sex, age, education, occupation, marital status) and geographical (Regions, urban/rural residence) features and the associated factors with adequate consumption, using the national baseline data.

## Methods

### Study design

A secondary cross-sectional analysis was performed using data from the first WHO STEPS [10] survey conducted in 2013 in Burkina Faso. All methods were carried out in accordance with relevant guidelines and regulations. The protocol of the STEPS survey was approved by the Ethics Committee for Health Research of the Ministry of Health of Burkina Faso (deliberation No: 2012–12092; December 05, 2012). Written informed consent was obtained from each participant in the study.

This STEPS survey is a recommended tool for surveillance of chronic diseases and their risk factors in WHO member countries. The survey is a standardized method to collect, analyse and disseminate data. It is a sequential process that starts with gathering key information about risk factors with a questionnaire; subsequently, simple physical measurements and blood samples for biochemical analysis are collected. The WHO STEPS includes a representative sample of the study population, which allows the results to be generalizable to the entire population.

### Study population

The study population was adults of both sexes aged 25 to 64 years who had been living in Burkina Faso for at least six months on the day of the survey.

### Sample size, data collection and participants included in the analyses

The total sample size calculation and the data collection process throughout the country have been described elsewhere [16]. The National Institute for Statistics and Demography (Institut National de la Statistique et de la Démographie, INSD) of Burkina Faso provided maps and data on enumeration areas (EAs) and their number of households which informed the representative sampling process. The INSD used data from the latest *General Census of Population and Housing* (2006) and updated in 2010 during the Demographic and Health Survey in Burkina Faso to define the EAs or clusters. More details on the EAs can be found elsewhere [17]. The sample size calculation in the WHO STEPS non-communicable disease risk factor survey was based on the prevalence of hypertension (primary outcome) and only a brief description will be presented for this secondary data analysis study. The survey enrolled adults aged 25–64 years, based on a calculated sample size, large enough to allow subgroup comparisons. The estimated sample size, based on an assumed prevalence of hypertension of 29.4, 5% precision, a design effect of 1.5 and 20% non-response, was 4785, and was rounded up to 4800. The sample was weighted by sex, age group, and rural/urban residence [16, 18].

A stratified three-stage cluster proportional to the size sampling was used to select participants. The sample was stratified to provide adequate representation of both rural and urban residence. An excel spread sheet was used to draw households from each selected cluster. One individual aged 25–64 years was randomly selected from each household using the Kish method [19].

The data collection team consisted of supervisors and interviewers. The supervisors were statisticians, epidemiologists and clinicians. The interviewers were nurses and medical students at the end of their training paths and who had proven experience in population surveys. The field staff was trained to collect the data using standard tools and methods. They were trained over a period of five days and participated in a field pre-test of the study instruments. Data were collected using a questionnaire and physical measurements. Data collection was conducted from 3 September to 24 October 2013. The data were collected using standardized WHO STEPS questionnaires input into laptop computers. Household sociodemographic information was recorded via face-to-face interviews in the language spoken by the participant after blood pressure and anthropometric measurements were collected.

After data collection, 105 individuals were not eligible or had invalid data regarding sex, 10 had missing data on marital status or education level and 283 on either fruits and vegetables. In total, 4402 participants were included in the analyses.

### Variables of interest extracted from the STEPS survey database

The participants’ demographic variables included gender, residence (rural, urban), age (25–64 years), marital status [grouped into i) married or cohabitating, ii) single], education level [grouped into i) no formal schooling, ii) primary school and iii) secondary or higher], and occupation [grouped into i) public or private employees with formal income or, ii) others i.e., without or unknown income, such as students, self-employed, housekeepers or unemployed]. The survey included details on FV intake. Data included the number of days (frequency) the respondent ate FV in a typical week, and on those days, how many servings (quantity) they ate. Servings were determined based on response after showing pictorial show cards (for uncooked items) or measuring cups (cooked items). The total typical daily intake of ≥ 5 FV was considered to be an adequate consumption. Despite the focus on adequate consumption, it is also relevant for public health considerations, to specify the consumption of at least 3 servings of fruits and at least 3 servings of vegetables [20, 21].

### Country’s climatic and urbanization features

**Fig. 1:**
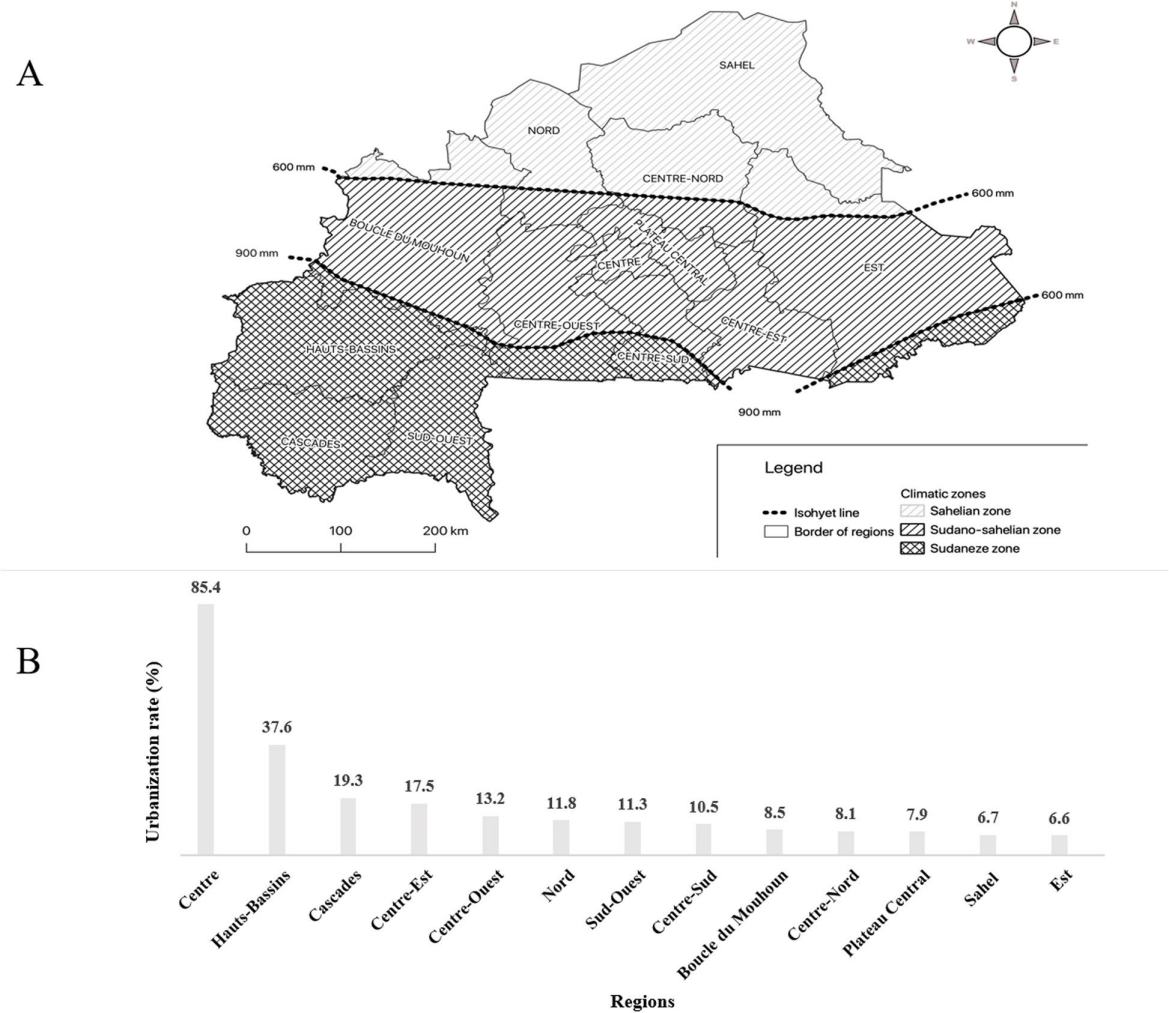
The Burkina Faso 13 administrative Regions with the limits of its three climatic zones (1.A) and the urbanization rates of Regions (1.B)

The climatic specificities and/or urbanisation features may influence food consumption habits [22, 23]. The country has a sub-Sahelian climate, the rainy season usually lasts from June until October [24]. The period November until February is considered the cool dry season, while the period March until May is called the hot dry season [24]. The country is characterized by three climatic zones [25]: i) the Sudanian zone to the South, ii) the Sudano-Sahelian zone going from East to West and iii) the Sahelian zone to the North (Fig. 1A). The Sudano-Sahelian zone is much larger than the other two climatic zones. Burkina Faso is divided into 13 administrative Regions (Fig. 1), each with a specific level of urbanization (Fig. 1B). The national mean rate is 23.3% (minimum = 6.6%, maximum = 85.4%) [17]. The “Centre” Region includes the political capital (Ouagadougou, with 46.4% of the country's urban dwellers) while the economic capital (Bobo-Dioulasso with 15.4% of the country's urban dwellers) [17] is in the “Hauts-Bassins” Region; and they have the higher rate of urbanisation (Fig. 1B).

The three climatic zones do not usually have identical rainy season lengths or rainfall patterns. There is no perfect fit (or congruence) between the country climatic map and administrative subdivision and thus, some administrative Regions are included in different climatic zones. The Fig. 1 provides an illustration.

### Categorization the 13 Regions using the national levels of the FV intake

The quartiles of the FV consumption were derived using the national/overall levels, and the Regions’ proportions of the people included in each quartile were determined. The first and the fourth quartiles respectively indicated the lowest and highest levels of the FV intake.

### Statistical analyses

StataCorp™ Stata Statistical Software for Windows (Version 14.0, College Station, Texas, United States of America) was used to analyse the data. The quantitative variables are expressed as the means ± standard deviations, and the qualitative variables are expressed as percentages (%) with 95% confidence intervals (CIs). Student’s t and anova tests were used to compare quantitative variables, and the chi-square or the Fishers exact tests were used to compare categorical variables. As performed elsewhere [13, 26], logistic regressions’ analyses were conducted to identify sociodemographic factors associated with the typical daily consumption of at least three servings of fruits, of at least three serving of vegetables and adequate FV consumption, using a progressive elimination of factors by decreasing the order of significance, i.e., with high level of the *p*-value. For all analyses, a *p*-value less than 0.05% was considered significant.

## Results

The sample was made up of 2307 (52.4%) females and the mean age was 38.3 ± 11.1 years. The participants were predominantly rural residents (79.4%) and illiterates (77.1%) (Table 1). The overall mean number of FV intake was 1.4 ± 1.9 (Supplemental Table 2).

**Table 1:** Sociodemographic characteristics, and fruit and/or vegetable (FV) consumption description, in the sample (*N* = 4402)

	**Overall**		**Ate ≥ 3 servings of fruits**				**Ate ≥ 3 servings of vegetables**				**Adequate FV consumption**			
	*N* = 4402													
	n	%	n	%	CI	*p*-value	n	%	CI	*p*-value	n	%	CI	*P*
**Residence**						0.45				0.249				0.011
**- Rural area**	3493	79.4	149	4.3	3.6–5.0		52	5.7	4.3–7.4		192	5.5	4.8–6.3	
**- Urban area**	909	20.6	33	3.6	2.5–5.1		237	6.8	6.0–7.7		31	3.4	2.3–4.8	
**Sex**						0.08				0.02				0.037
**- Male**	2095	47.6	75	3.6	2.8–4.5		118	5.6	4.7–6.7		91	4.3	3.5–5.3	
**- Female**	2307	52.4	107	4.6	3.8–5.6		171	7.4	6.4–8.6		132	5.7	4.8–6.7	
**Age range (years)**						0.30				0.66				0.41
**- 25–34**	2006	45.6	83	4.1	3.3–5.1		127	6.3	5.3–7.5		90	4.5	3.6–5.5	
**- 35–44**	1100	25.0	44	4.0	2.9–5.3		72	6.5	5.2–8.2		64	5.8	4.5–7.4	
**- 44–54**	786	17.8	40	5.1	3.7–6.9		50	6.4	4.8–8.3		42	5.3	3.9–7.2	
**- 55–64**	510	11.6	15	2.9	1.7–4.8		40	7.8	5.7–10.5		27	5.3	3.5–7.6	
**Marital status**						0.40				0.006				0.33
**- Married/cohabitating**	3826	86.9	20	4.2	3.6–4.9		236	6.2	5.4–7.0		189	4.9	4.3–5.7	
**- Single**	576	13.1	162	3.5	2.1–5.3		53	9.2	6.9–11.9		34	5.9	4.1–8.2	
**Occupation**						0.07				0.24				0.61
**- Employees with formal income**^a^	252	5.7	16	6.3	3.7–10.1		21	8.3	5.2–12.4		11	4.4	2.2–7.7	
**- Others**^b^	4150	94.3	166	4.0	3.4–4.6		268	6.5	5.7–7.2		212	5.1	4.5–5.8	
**Education level**						0.35				0.019				0.81
**- No formal education**	3394	77.1	135	4.0	3.3–4.7		205	6.0	5.3–6.9		168	5.0	4.2–5.7	
**- Primary school**	688	15.6	29	4.2	2.8–6.0		53	7.7	5.8–10.0		38	5.5	3.9–7.5	
**- Secondary or more**	320	7.3	18	5.6	3.4–8.7		31	9.7	6.7–13.5		17	5.3	3.1–8.4	

^a^Workers with formal monthly salary in the public or private sectors

^b^Others: Self-employed, house maker, jobless, students; *CI* Confidence interval at 95%

Almost a quarter (27.2% and 21.5% in rural and urban area respectively, *p* = 0.0001) of overall participants had not consumed any FV in the typical day (Supplemental Tables 1 & 2) and the prevalence of a typical daily consumption of at least three servings was 4.1% (95% CI: 3.6–4.8) for fruits and 6.6% (95% CI: 5.9–7.3) for vegetables (Table 2). The overall prevalence of adequate FV consumption was 5.1% (CI: 4.4–5.8) and was higher for the rural participants (5.5% vs 3.4% in the urban, *p* = 0.011) or the women (5.7% vs 4.3% in men, *p* = 0.037) (Table 1). Between the Burkina Faso 13 Regions, there was a significant wide range in the means’ numbers of the consumed FV (from 0.3 ± 0.4 to 3.0 ± 3.7; Supplemental Table 2) as well as in the prevalences of adequate FV intake (from 0.0% to 25.0%; Table 2). In the “Centre-Ouest” and “Nord” Regions, it was meet the higher prevalence of adequate FV consumption (19% and 25% respectively), as well as the higher mean number of consumed FV (2.6 ± 2.9 and 3.0 ± 3.7 respectively; Supplemental Table 2). To better understand these trends, we calculated the prevalence of adequate FV in these two regions (*N* = 639) in comparison with the rest of the regions (*N* = 3763). For these two Regions, the prevalence of adequate FV intake was 22.4% (CI: 19.2–25.8), while for the rest, the prevalence was significantly lower (2.4%; CI: 1.9–2.9), *p* = 0.0001. When categorizing the Regions’ participants according to the quartiles derived from the national number of the consumed FV, the two Regions (“Centre-Ouest” and “Nord”) had the higher proportion (about 50%) of their participants in the fourth quartile (high level) of consumption (Supplemental Figure).

**Table 2:** Prevalence of those who ate in the typical day, at least 3 servings of fruits, at least 3 serving of vegetables, and adequate consumption, by the country Regions

Regions	Number of participants	Ate at least 3 servings of fruits			Ate at least 3 servings of vegetables			Ate at least five servings (adequate) of FV		
	**N**	**n**	**%**	**95% CI**	**n**	**%**	**95% CI**	**n**	**%**	**95% IC**
**Centre**	533	9	1.7	0.8–3.2	24	4.5	2.9–6.6	11	2.1	1.0–3.7
**Est**	346	5	1.4	0.5–3.3	13	3.8	2.0–6.3	6	1.7	0.6–3.7
**Centre-Est**	384	1	0.3	< 0.0–1.4	16	4.2	2.4–6.7	7	1.8	0.7–3.7
**Centre-Sud**	216	4	0.2	0.5–4.7	7	3.2	1.3–6.6	9	4.2	1.9–7.8
**Centre-Nord**	429	19	4.4	2.7–6.8	20	4.6	2.9–7.1	10	2.3	1.1–4.2
**Sahel**	303	1	0.3	> 0.0–1.8	37	12.2	8.7–16.4	9	3.0	1.4–5.6
**Plateau Central**	236	1	0.4	> 0.0–2.3	0	0.0	–-	0	0.0	–-
**Cascades**	152	15	9.9	5.6–15.8	12	7.9	4.1–13.4	10	6.6	3.2–11.8
**Sud-Ouest**	214	5	2.3	0.8–5.4	3	1.4	0.3–4.0	3	1.4	0.3–4.0
**Boucle du Mouhoun**	467	13	2.8	1.5–4.7	18	3.9	2.3–6.0	11	2.4	1.2–4.2
**Centre-Ouest**	307	47	15.3	11.5–19.8	53	17.3	13.2–22.0	60	19.5	15.3–24.4
**Nord**	332	50	15.1	11.4–19.4	84	25.3	20.7–30.3	83	25.0	20.4–30.0
**Hauts-Bassins**	483	12	2.5	1.3–4.3	2	0.4	0.1–1.5	4	0.8	0.2–2.1
**Total/National**	4402	182	4.1	3.6–4.8	289	6.6	5.9–7.3	223	5.1	4.4–5.8

Using the the χ2 test to compare the prevalences of the adequate FV intake between these Regions, *p*-value was also 0.0001. For the two Regions of “Centre-Ouest” and “Nord”, the pooled prevalence of adequate FV intake was 22.4% (95% CI: 19.2 – 25.8), while for the other eleven Regions, it was significantly lower, 2.4% (95% CI: 1.9–2.9), *p* = 0.0001. Those who ate at least three servings of fruits among the participants of these two regions (“Centre-Ouest” and “Nord”) represented 15.2% (95% CI: 12.5 – 18.2) while 2.3% (95% CI: 1.8 – 2.8) for those living in the other eleven Region, *p* = 0.0001. Those who ate at least three servings of vegetables among the participants of these two regions (“Centre-Ouest” and “Nord”) represented 21.4% (95% CI: 18.3 – 24.8) while 4.0% (95% CI: 3.4 – 4.7), *p* = 0.0001 for people living in the other eleven Regions

*CI* Confident interval

In contrast to the higher urbanisation rate for the administrative Regions of "Centre" and "Hauts-Bassins" (Fig. 1B), there was a low level of adequate FV consumption (2.1% and 0.8% respectively) (Table 2).

The Table 3 summarizes the results from logistic regressions reporting that no socio-demographic factor was significantly associated with the consumption of at least three servings of fruit, while the rural residence, female gender and being educated were significantly associated with the consumption of at least three servings of vegetables. The two sociodemographic factors significantly associated with the adequate FV consumption were living in rural area (aOR = 1.66, CI:1.13–2.45, *p* = 0.011) and the female gender (aOR = 1.34; CI: 1.02–1.77, *p* = 0.033) (Table 3).

**Table 3:** Sociodemographic factors’ associations with fruit and/or vegetable intake (in the typical day) in logistic regression

	**Multivariable analysis**								
	**Ate at least 3 fruits**			**Ate at least 3 vegetables**			**Adequate intake**		
	aOR	95% CI	*p*-value	aOR	95% CI	*p*-value	aOR	95% CI	*p*-value
**Residency**									
**- Urban area (Ref)**	1			1			1		
**- Rural**	1.43	0.93–2.17	0.10	1.77	1.24- 2.54	0.002	1.66	1.13–2.45	0.01
**Gender**									
**- Male (Ref)**	1			1		1			
**- Female**	1.36	> 0.99–1.84	0.050	1.45	1.13- 1.85	0.003	1.34	1.02–1.77	0.033
**Age range (years)**									
**- 25–34**	1			1			1		
**- 35–44**	0.98	0.67–1.42	0.92	1.12	0.83–1.51	0.47	1.39	> 0.99–1.94	0.050
**- 44–54**	1.26	0.86–1.86	0.24	1.10	0.78–1.55	0.56	1.30	0.89–1.90	0.18
**- 55–64**	0.75	0.43–1.31	0.31	1.40	0.96–2.05	0.80	1.33	0.84–2.07	0.22
**Marital status**									0.18
**- Married/cohabitating (Ref)**	1			1			1		
**- Singles**	0.81	0.50–1.32	0.40	1.55	1.12–2.14	0.008	1.30	0.88–1.92	
**Occupation**									0.66
**- Others**^b^**(Ref)**	1			1			1		
**- Employees with formal regular income**^a^	1.63	0.96–2.76	0.072	1.04	0.58–1.88	0.89	0.84	0.39–1.83	
**Education level**									
**- No formal education**	1			1			1		
**- Primary school**	1.19	0.77–1.84	0.80	1.49	1.07–2.07	0.017	1.40	0.95–2.00	0.093
**- Secondary or more**	1.43	0.71–2.89	0.99	2.24	1.42–3.54	0.001	1.72	0.97–3.04	0.063

Whatever, with assumptions to control for confounders in the multivariable logistic regressions analyses with backward elimination:

- When only the independent factors statistically significant at the *p* < 0.25 level in bivariate analyses were included in the multivariate model, the final results from each multivariable logistic regression remained unchanged

- When only the factors statistically significant at the *p* < 0.05 level in bivariate analyses were included in the multivariate model as performed elsewhere [13, 26], the unique change in the final results was the absence of the independent variable “residency”, among the significantly associated variables with the dependent factor “ate at least three servings of vegetables”. Indeed, with a *p* = 0.249 at bivariable analysis, it (“residency”) cannot be included in the final model

^a^Workers with formal monthly salary in the public or private sectors

^b^Others: Self-employed, house maker, jobless, students); *CI* Confidence interval at 95%, *cOR* Crude odds-ratio, *aOR* Adjusted Odds-ratio

## Discussion

Except for the Regions of “Centre-Ouest” and “Nord”, prevalence of adequate consumption of FV was very low in the other eleven Regions of Burkina Faso, even it slightly increased in female gender and rural people.

### Overall prevalence

The overall prevalence of adequate FV consumption we found in Burkina Faso (5.1%) was low as it was observed in ASS countries by others authors using the STEPS or national data, i.e., the range from 1 to 8% in three other ASS countries (Ethiopia, Mozambique, Kenya [27–29]) or in Egypt and India [30, 31]. However, the higher prevalence range from 13 to 20% was found in three SSA countries (Uganda, Benin, Rwanda [32–34]) and in Bangladesh [35] and a better level of 26% to 30% was reported for Ghana, Nigeria [36, 37] and Thailand [38]. In LMICs, seasonal availability of some specific products source of micro-nutrients was correlated with some depletions [39, 40], the prevalence of vitamin A deficiency increased 1.6 (1.5–1.8) times higher in the rainy season compared to the dry, and varied up to twofold between regions in Guinea-Bissau [41]. The STEPS survey was held from September to October, period included in the rainy season period in Burkina Faso while mango (for example), a widespread fruit (in southwest of Burkina Faso) [42] and source of the Beta-carotene was not available by the survey time, and some authors reported low rate of the dietary diversity score or nutrient adequacy ratio by the rainy period [43].

### Disparity in prevalence (0.0% to 25.0%) of adequate FV intake between Regions

There is a low rainfall in the wide area of Burkina Faso (Fig. 1A) a Sahelian and vulnerable country [22, 23], and almost all plant species yielding FV do not provide products at all times or at each month of the year. During the rainy season, cereal farming is a priority for the country, while some specific localities undergo for off-season production of crops [44], that may include tomatoes, green bean, salad lettuce, potato leaves etc. during dry season. Burkina Faso is also facing a major challenge in terms of storage, processing, warehousing and distribution of FV, the perishable foodstuffs [45, 46]. Throughout the country, the variability in the seasonal availability of food and especially the FV, strongly influences the fluctuation in the household dietary diversity or individual consumption of FV [47]. There are different ethnic groups in Burkina Faso, potentially with specific socio-cultural perceptions on certain species of FV and their consumption and the habit for FV consumption as a cultural heritage in relation to the psycho-socio-cultural considerations of food choice should not be neglected [48, 49]. Our study indicates that efforts to promote The FV consumption in all country regions and provides a specific dashboard (Tables 2 & Supplemental data) for public health interventions throughout the country.

### High rates of urbanisation while low level of adequate intake

In the specific Regions of "Centre" and "Hauts-Bassins" which involved Burkina Faso political and economic capitals and the 61,8% of the country's urban dwellers [17], there was an alarming low prevalence (2.1% and 0.8%, respectively) of adequate FV consumption (Table 2). Moreover, urban residence was associated with decreased FV intake (Table 3) and was a major concern. Indeed, LMICs including SSA countries are undergoing nutrition [50, 51] and epidemiological [52, 53] transitions with surprising widespread of cardiovascular risk factors and diseases, strongly correlated with the urbanization process [54, 55]. Public health measures should urgently target the urban people in Burkina Faso.

### Low rates of urbanisation with increased level of adequate intake

The urbanization rate in the Regions of “Centre-Ouest” and “Nord” Regions was low (13.2% and 11.8% respectively) [17] (Fig. 1B) while a respective prevalence of adequate FV intake was 25.0% and 19.5% (Table 2). These regions are not the best-watered in Burkina Faso either (Fig. 1A). This could indicate a wider availability of FV in these two regions by the times of the STEPS survey, with greater compliance for consumption, or a habit of direct consumption of plant products. This may also support the association between rural residency and adequate FV intake (Table 3). Such behaviour should be encouraged and reinforced, while further studies should highlight benefits of this consumption in these areas, probably by comparing the levels of metabolic abnormalities (dyslipidaemia, obesity) between these regions of high FV intake and the others. This statement is also supported by the results in Supplemental Figure (the best adherence to the FV consumption in these Regions). The value of the FV consumption did not be well known by the general population [56] and thus, evidence on the potential health outcomes should be vulgarised in the Burkinabè communities in order to impulse engagement for the FV consumption.

### Female gender, favourable factors to the adequate FV intake

This association we found (Table 3) was similar to the report in Bangladesh [35] and Thailand [38]. Overweight/obesity more frequently affected Burkinabè women (overall prevalence of 19%, purchasing 44% in urban area [57]), and this problem could connect women with healthcare workers providing them with healthy advices driving to the better compliance to the FV consumption in overweight and obese adults [56]. Since the usefulness of the family-based approaches to cardiovascular health promotion or risk reduction [58, 59] and the nutrition education promoting FV consumption [56] is obvious, it should be initiated and women could potentially play the prominent role.

### Limitations

The study design was cross-sectional, and the survey was held from September to October and could not reflect the consumption level during the other months of the year. The optimum approach to collecting accurate food consumption data is not obvious, and the brief frequency questionnaire on FV consumption may have information biases and omissions, as this method also depends on participants' memory. The representative sample was calculated by using the prevalence of hypertension, included only adults aged 25 years and over, without the 15- to 24-year-olds, a very significant group in Burkina Faso. Data on the socio-economic level of participants would also have allowed us to better understand the level of FV intake [38]. There was no analysis of food regimens (un-available data) with regards to the level of the FV intake, that could show correlations and provide a better view and understanding of the consumption [13] and future studies should take this into account. The rainfall level by region and the regional production in FV at the moment of the survey as well as the exported and imported quantities of FV should be relevant parameters to be included in our analysis. While these first nationally-representative data from 2013 may no longer reflect the current situation, they provide a baseline that can be compared with future WHO STEPS survey data.

## Conclusion

Except for the Regions of “Centre-Ouest” and “Nord” of Burkina Faso, the prevalence of adequate consumption of FV was very low in its other eleven Regions. The climatic specificities including duration and alternance in dry and rainy seasons with the seasonal availability of the FV should be considered. Evidence on the potential health outcomes of FV consumption for the people residing in high intake areas (Regions of “Centre-Ouest” and “Nord”) should be highlighted and vulgarised within Burkinabè communities. This study indicates that efforts to improve consumption should be made in all regions of the country, and identifies the priority specific regions for intervention. These data also provide a dashboard for health stakeholders throughout the country. Measures to increase FV intake are urgent for the urban people while a prominent role could be assigned to women in the framework of the implementation of the family-based approaches and the nutrition education promoting FV consumption. The future survey using the STEPS method should allow assessment of the potential change in the consumption level.

### Supplementary Information

**Additional file 1: Supplemental Table 1.** Mean number of the consumed fruit and/or vegetables, prevalence of those did not consume any fruit or vegetable, and inadequate consumption in the total sample (*N* = 4402). **Supplemental Table 2.** Mean number of the consumed fruit and/or vegetable (FV), prevalence of those did not consume any fruit or vegetable FV, and inadequate consumption by country Region. **Supplemental Figure.** Proportion of people in each quartile (Q) of the number of consumed fruit and/or vegetables for each Region of the country and at the national level.

## Data Availability

The database of the STEPS survey used for this secondary analysis is available at the Ministry of Health of Burkina Faso and can be requested from bicababrico78@gmail.com.

## Abbreviations

aOR: Adjusted odds ratios
CI: Confidence interval
cOR: Crude odds ratio
FV: Fruits and/or vegetables
LMICs: Low and middle-income countries
NCDs: Non-communicable diseases
OR: Odds ratio
SSA: Sub-Saharan Africa
STEPS: Stepwise approach to surveillance
US: United States
WHO: World Health Organization

## References

[1] Gheorghe A, Griffiths U, Murphy A, Legido-Quigley H, Lamptey P, Perel P (2018). The economic burden of cardiovascular disease and hypertension in low- and middle-income countries: a systematic review. BMC Public Health.

[2] Mudie K, Jin MM, Tan null, Kendall L, Addo J, Dos-Santos-Silva I (2019). Non-communicable diseases in sub-Saharan Africa: a scoping review of large cohort studies. J Glob Health.

[3] Aune D, Giovannucci E, Boffetta P, Fadnes LT, Keum N, Norat T (2017). Fruit and vegetable intake and the risk of cardiovascular disease, total cancer and all-cause mortality-a systematic review and dose-response meta-analysis of prospective studies. Int J Epidemiol.

[4] Lee M, Lim M, Kim J (2019). Fruit and vegetable consumption and the metabolic syndrome: a systematic review and dose-response meta-analysis. Br J Nutr.

[5] Picasso MC, Lo-Tayraco JA, Ramos-Villanueva JM, Pasupuleti V, Hernandez AV (2019). Effect of vegetarian diets on the presentation of metabolic syndrome or its components: a systematic review and meta-analysis. Clin Nutr.

[6] Hartley L, Igbinedion E, Holmes J, Flowers N, Thorogood M, Clarke A, et al. Increased consumption of fruit and vegetables for the primary prevention of cardiovascular diseases. Cochrane Database Syst Rev. 2013;:CD009874. 10.1002/14651858.CD009874.pub2 PMC6464871 23736950

[7] Shin JY, Kim JY, Kang HT, Han KH, Shim JY (2015). Effect of fruits and vegetables on metabolic syndrome: a systematic review and meta-analysis of randomized controlled trials. Int J Food Sci Nutr.

[8] Cavallo DN, Horino M, McCarthy WJ (2016). Adult intake of minimally processed fruits and vegetables: associations with cardiometabolic disease risk factors. J Acad Nutr Diet.

[9] Adoukonou T, Yahouédéou B, Agbétou M, Hountada H, Choki B, Kossi O (2020). Prevalence of stroke survivors in Parakou in northern Benin: a door-to-door community survey. Rev Neurol (Paris).

[10] Bonita R, Winkelmann R, Douglas KA, de Courten M, McQueen DV, Puska P (2003). The WHO Stepwise Approach to Surveillance (Steps) of Non-Communicable Disease Risk Factors. Global Behavioral Risk Factor Surveillance.

[11] Alwan A, MacLean DR, Riley LM, d’Espaignet ET, Mathers CD, Stevens GA (2010). Monitoring and surveillance of chronic non-communicable diseases: progress and capacity in high-burden countries. Lancet.

[12] Mokdad AH, Stroup DF, Giles WH, Behavioral Risk Factor Surveillance Team (2003). Public health surveillance for behavioral risk factors in a changing environment. Recommendations from the Behavioral Risk Factor Surveillance Team. MMWR Recomm Rep.

[13] Singh JK, Acharya D, Gautam S, Adhikari M, Park J-H, Yoo S-J (2019). Socio-Demographic and Diet-Related Factors Associated with Insufficient Fruit and Vegetable Consumption among Adolescent Girls in Rural Communities of Southern Nepal. Int J Environ Res Public Health.

[14] Clary C, Lewis DJ, Flint E, Smith NR, Kestens Y, Cummins S (2016). The Local Food Environment and Fruit and Vegetable Intake: A Geographically Weighted Regression Approach in the ORiEL Study. Am J Epidemiol.

[15] Janda KM, Ranjit N, Salvo D, Nielsen A, Kaliszewski C, Hoelscher DM (2022). Association between Fresh Fruit and Vegetable Consumption and Purchasing Behaviors, Food Insecurity Status and Geographic Food Access among a Lower-Income, Racially/Ethnically Diverse Cohort in Central Texas. Nutrients.

[16] Soubeiga JK, Millogo T, Bicaba BW, Doulougou B, Kouanda S (2017). Prevalence and factors associated with hypertension in Burkina Faso: a countrywide cross-sectional study. BMC Public Health.

[17] Ministère de l’économie et des finances du Burkina Faso. Recensement général de la population et de l’habitation de 2006 : Résultats définitifs. 2008. http://www.cns.bf/IMG/pdf/RGPH_2006.pdf. Accessed 10 Aug 2021.

[18] Diendéré J, Kaboré J, Bosu WK, Somé JW, Garanet F, Ouédraogo PV (2022). A comparison of unhealthy lifestyle practices among adults with hypertension aware and unaware of their hypertensive status: results from the 2013 WHO STEPS survey in Burkina Faso. BMC Public Health.

[19] Wiegand H, Kish L. Survey Sampling. John Wiley & Sons, Inc., New York, London 1965, IX + 643 S., 31 Abb., 56 Tab., Preis 83 s. Biom Z. 1968;10:88–9.

[20] He FJ, Nowson CA, MacGregor GA (2006). Fruit and vegetable consumption and stroke: meta-analysis of cohort studies. The Lancet.

[21] Miller V, Mente A, Dehghan M, Rangarajan S, Zhang X, Swaminathan S (2017). Fruit, vegetable, and legume intake, and cardiovascular disease and deaths in 18 countries (PURE): a prospective cohort study. Lancet.

[22] Sossou S, Igue CB, Diallo M (2019). Impact of Climate Change on Cereal Yield and Production in the Sahel: Case of Burkina Faso. SSRN Scholarly Paper.

[23] Nouaceur Z, Murarescu O (2020). Rainfall Variability and Trend Analysis of Rainfall in West Africa (Senegal, Mauritania, Burkina Faso). Water.

[24] Kaboré B, Kam S, Ouedraogo G, Bathiebo D (2017). Etude de l’évolution climatique au Burkina Faso de 1983 à 2012: cas des villes de Bobo Dioulasso. Ouagadougou et Dori AJES.

[25] Neya T, Neya O, Abunyewa AA (2018). Agroforestry parkland profiles in three climatic zones of Burkina Faso. Int J Biol Chem Sci.

[26] Peltzer K, Pengpid S (2015). Correlates of healthy fruit and vegetable diet in students in low-, middle- and high-income countries. Int J Public Health.

[27] Gelibo T, Amenu K, Taddele T, Taye G, Getnet M, Getachew T (2017). Low fruit and vegetable intake and its associated factors in Ethiopia: A community based cross sectional NCD steps survey. EJHD.

[28] Padrão P, Laszczyńska O, Silva-Matos C, Damasceno A, Lunet N (2012). Low fruit and vegetable consumption in Mozambique: results from a WHO STEPwise approach to chronic disease risk factor surveillance. Br J Nutr.

[29] Pengpid S, Peltzer K (2018). The prevalence and social determinants of fruit and vegetable consumption among adults in Kenya: a cross-sectional national population-based survey, 2015. Pan Afr Med J.

[30] Gadallah M, Megid SA, Mohsen A, Kandil S (2018). Hypertension and associated cardiovascular risk factors among urban slum dwellers in Egypt: a population-based survey. East Mediterr Health J.

[31] Tripathy JP, Thakur JS, Jeet G, Chawla S, Jain S, Prasad R (2016). Urban rural differences in diet, physical activity and obesity in India: are we witnessing the great Indian equalisation? Results from a cross-sectional STEPS survey. BMC Public Health.

[32] Guwatudde D, Mutungi G, Wesonga R, Kajjura R, Kasule H, Muwonge J (2015). The Epidemiology of Hypertension in Uganda: Findings from the National Non-Communicable Diseases Risk Factor Survey. PLoS ONE.

[33] Houehanou YCN, Lacroix P, Mizehoun GC, Preux P-M, Marin B, Houinato DS (2015). Magnitude of Cardiovascular Risk Factors in Rural and Urban Areas in Benin: Findings from a Nationwide Steps Survey. PLoS ONE.

[34] Nahimana M-R, Nyandwi A, Muhimpundu MA, Olu O, Condo JU, Rusanganwa A (2017). A population-based national estimate of the prevalence and risk factors associated with hypertension in Rwanda: implications for prevention and control. BMC Public Health.

[35] Karim MN, Zaman MM, Rahman MM, Chowdhury MAJ, Ahsan HAMN, Hassan MM (2017). Sociodemographic Determinants of Low Fruit and Vegetable Consumption Among Bangladeshi Adults: Results From WHO-STEPS Survey 2010. Asia Pac J Public Health.

[36] Atibila F, Dabo EO, Asamani JA, Adjei CA, Akugri FA, Attafuah PA (2018). Assessment of risk factors for hypertension in Dormaa municipality in Ghana using the World Health Organization STEPS approach. J Health Sci.

[37] Okpechi IG, Chukwuonye II, Tiffin N, Madukwe OO, Onyeonoro UU, Umeizudike TI (2013). Blood Pressure Gradients and Cardiovascular Risk Factors in Urban and Rural Populations in Abia State South Eastern Nigeria Using the WHO STEPwise Approach. PLoS ONE.

[38] Satheannoppakao W, Aekplakorn W, Pradipasen M (2009). Fruit and vegetable consumption and its recommended intake associated with sociodemographic factors: Thailand National Health Examination Survey III. Public Health Nutr.

[39] Sinha DP, Bang FB (1973). Seasonal variation in signs of vitamin-a deficiency in rural west Bengal children. The Lancet.

[40] Faber M, Laubscher R (2008). Seasonal availability and dietary intake of beta-carotene-rich vegetables and fruit of 2-year-old to 5-year-old children in a rural South African setting growing these crops at household level. Int J Food Sci Nutr.

[41] Danneskiold-Samsøe N, Fisker AB, Jørgensen MJ, Ravn H, Andersen A, Balde ID (2013). Determinants of vitamin a deficiency in children between 6 months and 2 years of age in Guinea-Bissau. BMC Public Health.

[42] Vayssieres J, Coulibaly O, Sinzogan A, Adandonon A, Dakouo D, Dabiré R (2012). Mango Cultivation in Burkina Faso.

[43] Nana CP, Brouwer ID, Zagré N-M, Kok FJ, Traoré AS (2005). Community Assessment of Availability, Consumption, and Cultural Acceptability of Food Sources of (PRO)Vitamin A: Toward the Development of a Dietary Intervention among Preschool Children in Rural Burkina Faso. Food Nutr Bull.

[44] Alaofè H, Burney J, Naylor R, Taren D (2016). Solar-Powered Drip Irrigation Impacts on Crops Production Diversity and Dietary Diversity in Northern Benin. Food Nutr Bull.

[45] Totobesola M, Delve R, Nkundimana JD, Cini L, Gianfelici F, Mvumi B (2022). A holistic approach to food loss reduction in Africa: food loss analysis, integrated capacity development and policy implications. Food Sec.

[46] Le Cotty T, Maître d’Hôtel E, Subervie J (2023). Inventory credit to enhance food security in Burkina Faso. World Dev.

[47] Somé JW, Jones AD (2018). The influence of crop production and socioeconomic factors on seasonal household dietary diversity in Burkina Faso. PLoS ONE.

[48] James D (2004). Factors influencing food choices, dietary intake, and nutrition-related attitudes among African Americans: Application of a culturally sensitive model. Ethn Health.

[49] Haghighian Roudsari A, Vedadhir A, Amiri P, Kalantari N, Omidvar N, Eini-Zinab H (2017). Psycho-Socio-Cultural Determinants of Food Choice: A Qualitative Study on Adults in Social and Cultural Context of Iran. Iran J Psychiatry.

[50] Popkin BM (1998). The nutrition transition and its health implications in lower-income countries. Public Health Nutr.

[51] Pedro JM, Brito M, Barros H (2018). Gender and socio-demographic distribution of body mass index: The nutrition transition in an adult Angolan community. J Public Health Afr.

[52] Bastien M, Poirier P, Lemieux I, Després J-P (2014). Overview of epidemiology and contribution of obesity to cardiovascular disease. Prog Cardiovasc Dis.

[53] Macia E, Cohen E, Boetsch G, Boetsch L, Cohen E, Duboz P (2017). Prevalence of obesity and body size perceptions in urban and rural Senegal: new insight on the epidemiological transition in West Africa. Cardiovasc J Afr.

[54] Popkin BM (1999). Urbanization, Lifestyle Changes and the Nutrition Transition. World Dev.

[55] Zeba AN, Yaméogo MT, Tougouma SJ-B, Kassié D, Fournet F (2017). Can Urbanization, Social and Spatial Disparities Help to Understand the Rise of Cardiometabolic Risk Factors in Bobo-Dioulasso? A Study in a Secondary City of Burkina Faso, West Africa. Int J Environ Res Public Health.

[56] Wagner MG, Rhee Y, Honrath K, Blodgett Salafia EH, Terbizan D (2016). Nutrition education effective in increasing fruit and vegetable consumption among overweight and obese adults. Appetite.

[57] Diendéré J, Kaboré J, Somé JW, Tougri G, Zeba AN, Tinto H (2019). Prevalence and factors associated with overweight and obesity among rural and urban women in Burkina Faso. Pan Afr Med J.

[58] Vedanthan R, Bansilal S, Soto AV, Kovacic JC, Latina J, Jaslow R (2016). Family-Based Approaches to Cardiovascular Health Promotion. J Am Coll Cardiol.

[59] Sawhney J, Madan K (2021). Family-based approach in cardiovascular risk reduction. Lancet Glob Health.

