# Supplementary material for: Geographical and sociodemographic disparities in fruit and vegetables consumption among adults in Burkina Faso: baseline results from the 2013 WHO STEPS survey
Source: BMC Public Health. 2023 Nov 14;23:2245. doi: 10.1186/s12889-023-17118-0 (PMC10644490; doi:10.1186/s12889-023-17118-0)
Supplement: Supplementary file 1 — Additional file 1: Supplemental Table 1. Mean number of the consumed fruit and/or vegetables, prevalence of those did not consume any fruit or vegetable, and inadequate consumption in the total sample (N = 4402). Supplemental Table 2. Mean number of the consumed fruit and/or vegetable (FV), prevalence of those did not consume any fruit or vegetable FV, and inadequate consumption by country Region. Supplemental Figure. Proportion of people in each quartile (Q) of the number of consumed fruit and/or vegetables for each Region of the country and at the national level. [file 12889_2023_17118_MOESM1_ESM.pdf]

Supplemental Data

Geographical and sociodemographic disparities in fruit and vegetables consumption among adults in Burkina Faso: baseline results from the 2013 WHO STEPS Survey

Jeoffray Diendéré, Jérôme Winbetouréfâ Somé, Jean Kaboré, Amadé Sawadogo, Estelle-Edith Dabiré, Ella Rakèta W. Compaoré, Athanase Millogo and Augustin Nawidimbasba Zeba

Supplemental Table 1: Mean number of the consumed fruit and/or vegetables, prevalence of those did not consume any fruit or vegetable, and inadequate consumption in the total sample (N = 4402).

	**Overall**	**Mean, standard deviation in total number of FV**			**Prevalence of those who did not consume any fruit or vegetable in the typical day**				**Prevalence of inadequate FV consumption**			
	N	Mean	Sd	p-value	N	%	CI	P-value	n	%	CI	P-value
**Residence**				0.40				0.0001				0.011
- **Rural area**	3493	1.5	1.7		951	27.2	25.8-28.7		3301	94.5	93.7-95.2	
- **Urban area**	909	1.4	2.0		114	12.5	10.5-14.9		878	96.6	95.2-97.7	
**Sex**				0.063				0.33				0.037
- **Male**	2095	1.4	1.8		493	23.5	21.7-25.4		2004	95.7	94.7-96.5	
- **Female**	2307	1.5	2.0		572	24.8	23.0-26.6		2175	94.3	93.3-95.2	
**Age range (years)**				0.87				0.22				0.41
- **25-34**	2006	1.4	1.8		463	23.1	21.3-25.0		1916	95.5	94.5-96.4	
- **35-44**	1100	1.5	2.0		262	23.8	21.3-26.4		1036	94.2	92.6-95.5	
- **44-54**	786	1.4	2.0		208	26.5	23.4-29.7		744	94.7	92.8-96.1	
- **55-64**	510	1.4	1.9		132	25.9	22.1-29.9		483	94.7	92.4-96.5	
**Marital status**				0.067				0.01				0.33
- **Married/cohabitating**	3826	1.4	1.9		958	25.0	23.7-26.4		3637	95.1	94.3-95.7	
- **Single**	576	1.6	2.1		107	18.6	15.5-22.0		542	94.1	91.8-95.9	
**Occupation**				0.0006				0.0001				0.61
- **Employees with formal income***	252	1.8	1.8		22	8.7	5.6-12.9		241	95.6	92.3-97.8	
- **Others ** **	4150	1.4	1.9		1043	25.1	23.8-26.5		3938	94.9	94.2-95.5	

**Education level**				0.0001				0.0001				0.81
- **No formal education**	3394	1.4	1.9		920	27.1	25.6-28.6		3226	95.0	94.3-95.8	
- **Primary school**	688	1.5	1.7		131	19.0	16.2-22.2		650	94.5	92.5-96.1	
- **Secondary or more**	320	2.0	1.8		14	4.4	2.4-7.2		303	94.7	91.6-96.9	

*: Workers with formal monthly salary in the public or private sectors; ** Others: Self-employed, house maker, jobless, students; CI: confidence interval at 95%.

Supplemental Table 2: Mean number of the consumed fruit and/or vegetable (FV), prevalence of those did not consume any fruit or vegetable FV, and inadequate consumption by country Region

Regions	Number of participants	Number of FV intake		Did not eat any fruit or vegetables			Inadequate FV intake		
	N	$\bar{X}$	SD	N	%	95% CI	N	%	95% CI
**Centre**	533	1.3	1.3	39	7.3	5.3-9.9	522	97.9	96.3-99.0
**Est**	346	1.8	1.3	52	15.0	11.4-19.2	340	98.3	96.3-99.4
**Centre-Est**	384	1.4	1.4	96	25.0	20.7-29.6	377	98.2	96.3-99.3
**Centre-Sud**	216	1.7	1.7	39	18.1	13.2-23.8	207	95.8	92.2-98.1
**Centre-Nord**	429	0.8	1.6	239	55.7	50.9-60.5	419	97.7	95.8-98.9
**Sahel**	303	1.1	1.7	156	51.5	45.7-57.2	294	97.0	94.4-98.6
**Plateau Central**	236	0.3	0.4	87	36.9	30.7-43.4	236	100	---
**Cascades**	152	1.8	2.0	9	5.9	2.7-10.9	142	93.4	88.2-96.8
**Sud-Ouest**	214	0.9	1.1	40	18.7	13.7-24.6	211	98.6	96.0-99.7
**Boucle du Mouhoun**	467	1.1	1.5	128	27.4	23.4-31.7	456	97.6	95.8-98.8
**Centre-Ouest**	307	2.6	2.9	46	15.0	11.2-19.5	247	80.5	75.6-84.7
**Nord**	332	3.0	3.7	63	19.0	14.9-23.6	249	75.0	70.0-79.6
**Hauts-Bassins**	483	1.1	1.1	71	14.7	11.7-18.2	479	99.2	97.9-99.8
**Total/National**	4402	1.4	1.9	1065	24.2	22.9-25.5	4179	94.9	94.2-95.6

Using the anova test to compare mean numbers of fruits and vegetables (FV) between the 13 Regions, the p-value was 0.0001, and when the using the the $\chi 2$ test to compare the prevalences of the inadequate FV intake between these Regions, p-value was also 0.0001. In the two Regions of "Centre-Ouest" and "Nord", prevalence of inadequate FV intake was 77.6% (95% CI: 74.2-80.8), while in the other eleven Regions, prevalence was significantly higher, 97.6% (95% CI: 97.4-98.3), p=0.0001. The mean number of consumed FV in the areas of high (Regions of "Centre-Ouest" and "Nord") and low (other eleven Regions) consumption was respectively 2.8 (±3.3) and 1.2 (±1.4), p=0.0001.

$\bar{X}$: mean number; **SD**: standard deviation, CI: confident interval.

18

Supplemental Figure

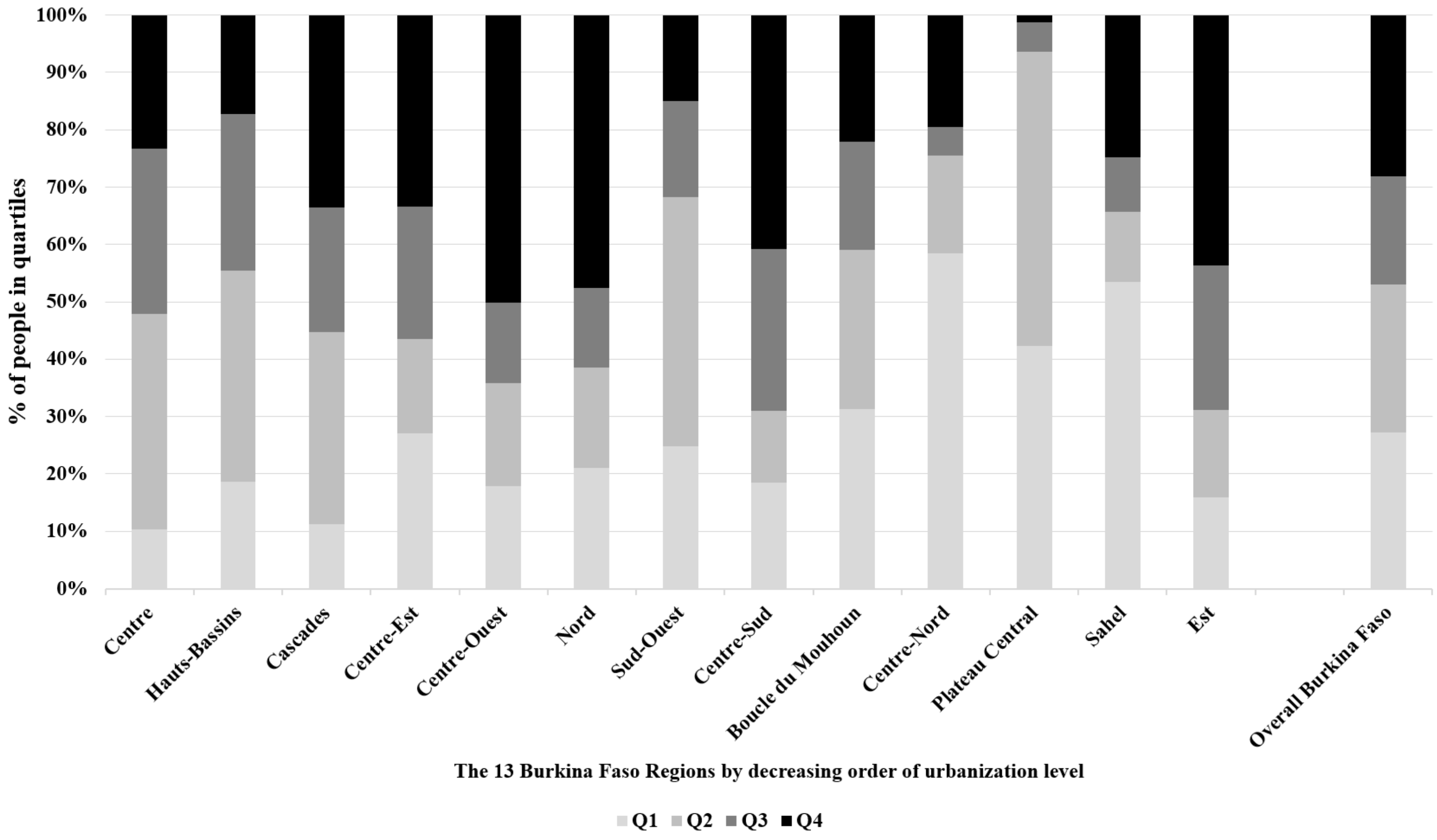

19

Supplemental Figure: Proportion of people in each quartile (Q) of the number of consumed fruit and/or vegetables for each Region of the country and at the national level.

The higher the quartile, the higher the intake.

The cut-offs for the quartile were derived from the national levels of consumption and were 0.142; 0.858 and 2.000 and the mean value of consumed FV were 0.02±0.04; 0.54±0.21; 1.35±0.27 and 3.67±2.30 within the first (Q1), second (Q2), third (Q3) and forth (Q4) quartiles respectively.
